# Development and Validation of a Micronutrients Knowledge, Attitudes, and Practices Questionnaire for Adolescents Using Psychometric Analysis

**DOI:** 10.7759/cureus.65628

**Published:** 2024-07-29

**Authors:** Priyanka Pareek, Aparna Thorat, Chethana Chandrasekar, Poonam Khanna, Rashmi Kulkarni

**Affiliations:** 1 Clinical Nutrition, Mahatma Gandhi Mission (MGM) School of Biomedical Sciences, Mahatma Gandhi Mission Institute of Health Sciences, Navi Mumbai, IND; 2 Community Medicine and School of Public Health, Postgraduate Institute of Medical Education and Research, Chandigarh, IND

**Keywords:** kap, nutrition education, micronutrients, health belief model, validation, adolescents

## Abstract

Introduction

The development of a questionnaire that measures knowledge, attitude, and practices towards micronutrients will help to develop nutrition intervention programs. The present study aimed to develop and assess the validity and reliability of a questionnaire on knowledge, attitude, and practices (KAP) on micronutrients in adolescents in India.

Methods

A total of 150 adolescent girls participated in the study. The literature was reviewed to formulate an initial draft of a questionnaire (122 items). Face and content validity were measured by participants and subject experts, respectively, and the content validity index was calculated. Construct validity was assessed using the principal component method of exploratory factor analysis. Internal consistency and test-retest reliability were determined by Cronbach's α value and interclass correlation coefficient correlations, respectively.

Results

The content validity index for all items except eight items from the practice subscale was satisfactory. Face validity results showed that participants understood all items. Exploratory factor analysis suggested a four-factor construct (perceived susceptibility and severity, perceived benefits, readiness to change, and perceived barrier) in the attitude subscale. Internal consistency for knowledge, attitude, and practice items were 0.980, 0.840, and 0.930, respectively. For knowledge and attitude items, interclass correlation coefficient correlation estimates ranged between 0.705 to 0.987 and 0.775 to 0.997, respectively, whereas for practice items, it ranged from 0.701 to 0.945. In the final questionnaire, 134 items consisting of 33 knowledge, 21 attitudes, and 80 practices (55 dietary practices and 25 other practices related to micronutrients) were included.

Conclusion

The results provided evidence of the validity and reliability of the questionnaire on micronutrients and that can be used to assess the knowledge, attitude, and practices on micronutrients in adolescents. Further studies in different diverse settings are recommended.

## Introduction

Vitamins and minerals collectively known as micronutrients, although required in small amounts, are essential for proper growth, development, and functioning of the human body. Micronutrient deficiency is defined as 'hidden hunger' and has an impact on health, resulting in low productivity and a cycle of malnutrition, underdevelopment, and poverty [1]. Infants and children, women of reproductive age, pregnant women, and the elderly are the most vulnerable populations to these deficiencies. The main factors associated with micronutrient deficiencies are lack of dietary diversity, poor mineral bioavailability because of antinutrients in plant-based diets, incidence of illness and disease, and increased physiological demand [2]. Micronutrient deficiencies affect one-third of the world’s population [3]. Around 0.5 percent of total deaths in India were attributed to nutritional deficiencies in India in 2016 [4]. The government of India has initiated numerous initiatives and programs in response to micronutrient malnutrition, such as fortification, micronutrient supplementation, diet diversification, and nutrition education to prevent the problem of micronutrient malnutrition [5]. However, the problem of malnutrition still exists in a large proportion of the population. Micronutrients are necessary throughout life, but the period of childhood and adolescence is crucial due to the rapid growth and development that occurs during this time. As per the National Family Health Survey 2019-2021 (NFHS-5), the prevalence of anemia was 59.1% in adolescent girls [6]. According to the Comprehensive National Nutrition Survey, 2016-2018, on adolescents, the prevalence of vitamin A and vitamin D deficiency was 16% and 24%, respectively. More than one-third of the adolescents (32% and 31%) had zinc and vitamin B12 deficiencies, respectively. About two-fourths of the adolescents (37%) had folate deficiency [7]. A multi-center cross-sectional study was conducted in urban school-going children and adolescents in India by Awasthi et al. (2022) among age groups of six to 11 and 12 to 16 years (2428 participants selected from 60 schools) and results showed that the prevalence of calcium, iron, vitamin D and vitamin B 12 deficiency was 59.9% and 49.4%, 39.7%, and 33.4%, respectively [8].

For addressing the micronutrient deficiencies, a more comprehensive, long-term sustainable approach is required. Diet diversification is considered a sustainable strategy that offers a package of micronutrients and nutrition education [9]. It is necessary to collect information on dietary patterns as well as local food availability, cost, and accessibility to enable everyone to buy and consume a variety of foods that can help prevent micronutrient deficiencies through diet diversification. Evidence-based interventions are required to bring about sustainable social and behavioral change in people regarding micronutrient deficiency. To change the behavior of people towards the consumption of micronutrient-rich foods and to promote dietary diversification, one has to understand the current knowledge, attitudes, and practices (KAP) prevalent in the community. KAP can be used in developing nutrition education intervention programs to improve the micronutrient status of the target population. The literature on the development of validated and reliable KAP tools on micronutrients is limited. Therefore, there is a dire need to develop and validate a KAP questionnaire specific to micronutrient status, which would not only facilitate the identification of prevailing knowledge gaps, attitudes, and behaviors related to micronutrient intake but also serve as a foundational resource for designing targeted interventions that address these specific needs.

## Materials and methods

Study design and participants

Ethical approval was obtained from the Institutional Ethics Committee, MGM Institute of Health Sciences, Approval no. MGMIHS/RES./02/2022/178. The study was carried out at a private school in Navi Mumbai, India. A total of 150 adolescent girls with an age range of 13-18 years were included. Before data collection, written and informed assent was obtained from all participants, and consent was obtained from their parents.

**Figure 1:**
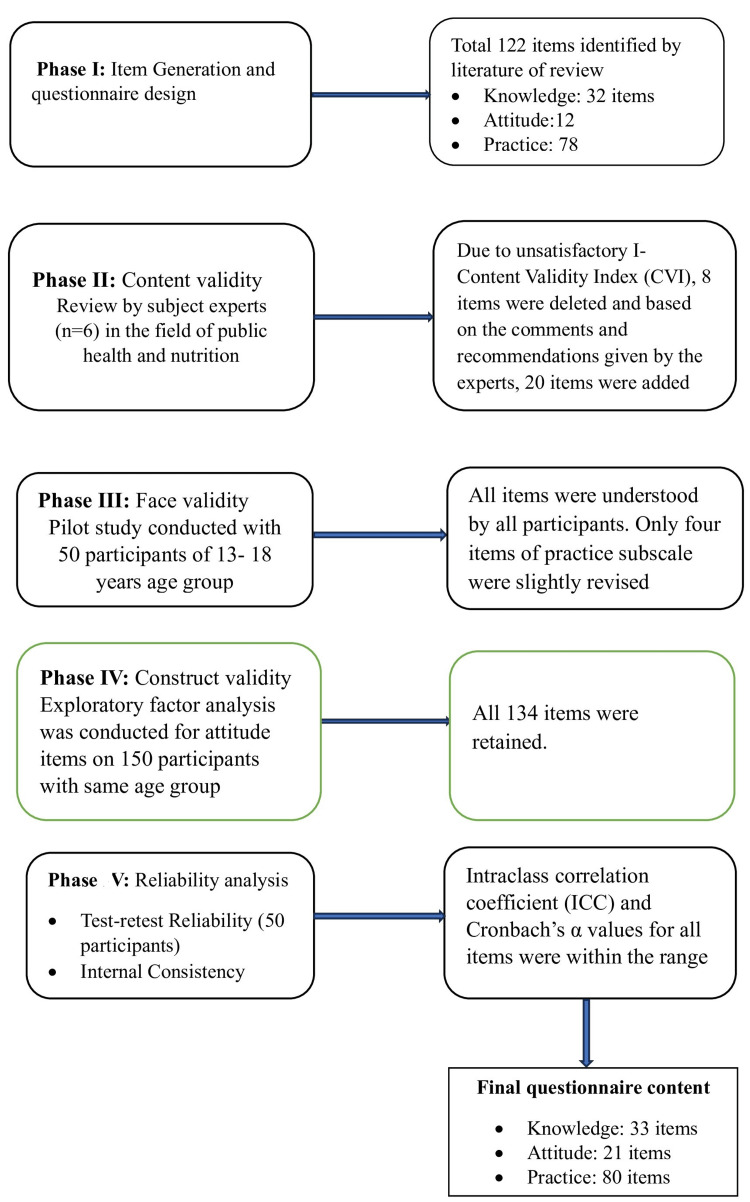
Flow chart of the questionnaire development process.

The development and validation of the questionnaire was based on five steps: Item development, assessing a face, content and construct validity, and reliability (Figure 1). 

Phase 1: Item Development

A comprehensive literature review was conducted to elucidate the primary understanding required for formulating the initial questionnaire. This process involved the exploration of scholarly articles about pertinent themes, employing targeted keywords including "nutrition knowledge," "attitude and practice," "adolescent nutrition," "micronutrients," "dietary assessments," "diet and micronutrient deficiencies," "factors affecting micronutrient deficiencies," and "menstruation and nutrition status." The search was conducted across renowned databases, such as PubMed, Cochrane, Scopus, and Google Scholar, and authoritative repositories, including those of the World Health Organization (WHO), Food and Agriculture Organization (FAO), and NFHS-5.

Initial draft of the questionnaire: Based on the results of the literature search, 122 items based semi-structured questionnaire on micronutrient knowledge (no. of items=32), attitude (no. of items=12), and practice (no. of items=78) was developed.

In the knowledge subscale, questions were included based on awareness about micronutrient sources, deficiency symptoms, causes of micronutrient deficiency, enhancers and inhibitors of absorption, and micronutrient supplementation. All questions were open-ended. The one correct answer to each question was given one mark, and if there were more than one correct answer, two marks were given. Each wrong answer or “don’t know” was given zero points.

The attitude subscale was based on Health Belief Model (HBM) constructs: perceived susceptibility and severity, perceived benefits, perceived barriers, and readiness to change [10]. All items (n=12) were rated on a three-point Likert scale: “Agree,” “Undecided,” and “Disagree”. For each statement, the responses “Agree,” “Undecided,” and “Disagree” were given as three, two, and one scores, respectively.

In the practice subscale, items on sunlight exposure, cooking practices, enhancers, inhibitors, supplementation (n=26), and dietary practices (n=52) were included. Dietary practice questions included participants’ frequency of consumption of micronutrient-rich foods in a month. Responses for the frequency of food items were assessed on a seven-point Likert scale with numeric scores of 0 to 6 (from “never” to “daily”). The next step was to assess the validity and reliability of the 122-item KAP questionnaire.

Phase II: Content Validity of the Questionnaire

The developed questionnaire was sent to six experts (two nutritionists, two researchers who had experience with the validation of the questionnaire, and two public health nutritionists) for content validation. The experts were asked to evaluate each item for relevancy and requested them to rate each item using a four-point Likert scale (1=not relevant, 2=somewhat relevant, 3=relevant, and 4=highly relevant). They were also asked to give comments. The content validity index (CVI) was calculated as the number of experts (who gave a score of 3 or 4 to each item for relevancy) divided by the total number of experts. An item with a CVI higher than 0.8 was retained and <0.8 CVI was removed [11].

Phase III: Face Validity

Subsample (n=50) participants were asked to evaluate the questionnaire to see whether the items were understandable and clear and also asked them to identify any difficult/ambiguous words or phrases.

Phase IV: Construct Validity

The underlying constructs of attitude items were assessed by the principal component method of exploratory factor analysis (EFA) using varimax rotations. EFA was performed on 150 participants who answered the questionnaire. The Kaiser-Meyer-Olkin (KMO) test was used for sampling adequacy (>0.6), and Bartlett’s test of sphericity (p≤0.05) was considered for the suitability of conducting factor analysis [12]. Eigenvalue (>1) and factor loadings >0.4 were assessed to determine the number of factors.

Phase V: Reliability

Internal consistency explains how well all the items in a test measure the same concept or construct. Internal consistency of the items in each subscale was assessed using Cronbach’s α value ≥0.7 [13].

The test-retest was evaluated using a single measurement, absolute agreement, and two-way mixed effects model and interpreted based on intraclass correlation coefficient (ICC), >0.70 as good reliability [14]. The questionnaire was administered twice on subsample (n=50 participants) at an interval of 15 days to see if the questionnaire was stable over time.

Statistical analysis

Data was analyzed using IBM® SPSS version 25 (SPSS, Chicago, IL, USA). Frequency and percentages were calculated for categorical variables. The mean and standard deviation values were calculated for continuous variables. Item analysis (Cronbach’s α and ICC’s) and exploratory factor analysis using principal component analysis and varimax rotation for construct validity were done on SPSS. For CVI estimation (to assess content validity), Microsoft Excel (Microsoft Corporation, Redmond, Washington, USA) was used.

## Results

A total of 150 participants were included in the pilot test to evaluate the validity, internal consistency, and test-retest reliability of the questionnaire. The mean age of the participants was 14.78 ± 1.40 years.

As shown in Table 1, the mean CVI score for relevance in the questionnaire was 0.882, whereas it ranged from 0.833 to 1 for knowledge and attitude items and from 0.5 to 1 for items in the practice subscale. After considering the points given to each item by expert panels, eight items were excluded from the practice subscale (the CVI of these items was less than 0.80). Based on the comments and recommendations given by the experts, one, nine, and 10 new items were added to the knowledge, attitude, and practice subscales, respectively, and also the wording and phrasing of some of the items were revised.

**Table 1:** Mean content validity index (CVI) of items in the subscale.

Subscale	No. of items	Mean CVI	Range
Knowledge	32	0.938	0.833-1
Attitude	12	0.858	0.833-1
Practices	78	0.863	0.5-1
All items	122	0.882	0.5-1

At the end of the content validity, the 134-item questionnaire consists of 33 knowledge, 21 attitudes, and 80 practice items (55 dietary practices and 25 other practices related to micronutrients).

Based on face validity results, all participants stated most of the items were easy to understand and clear. Only a few items in the practice subscale were slightly revised based on the participant’s response.

In the exploratory factor analysis, results showed that the KMO was adequate for attitude items (0.882), and Bartlett’s test of sphericity was found to be significant (χ^2^=5268.594, p<0.001). The principal component method was used to extract factors with an eigenvalue above 1, and this method extracted four dimensions in the attitude scale with 42.21%, 72.26%, 81.95%, and 86.71% of the variance explained in the first, second, third, and fourth dimensions, respectively. The first dimension included nine items that measured concepts of “perceived susceptibility and severity,” and dimension 2 (five items) was named “perceived benefits,” whereas dimensions 3 and 4 determined “readiness to change” (six items) and “perceived barrier” (one item) respectively (Table 2).

**Table 2:** Exploratory factor analysis of attitude subscale. Extraction method: principal component analysis, rotation method: varimax with Kaiser normalization, factor loading cut off >0.40. KMO: Kaiser-Meyer-Olkin.

Factors	Initial Eigenvalues			Extraction sums of squared loadings			Range of factor loadings
	Total	% of variance	Cumulative percentage	Total	% of variance	Cumulative percentage	
Perceived susceptibility	8.864	42.210	42.210	8.864	42.210	42.210	0.878-0.976
Perceived benefits	6.313	30.064	72.275	6.313	30.064	72.275	0.887-0.941
Readiness to change	2.032	9.674	81.949	2.032	9.674	81.949	0.590-0.923
Perceived barriers	1.000	4.764	86.713	1.000	4.764	86.713	0.986
KMO value	0.882						
Bartlett’s test of sphericity value	χ^2^=5268.594 (p<0.001)						

In the practice subscale, only food frequency items (55) were included to calculate the internal consistency. Other items (25) in the practice subscale were used to assess the qualitative information.

The total Cronbach’s α value for the knowledge, attitude, and practices subscales were 0.980, 0.840, and 0.930, respectively (Table 3). All items had Cronbach’s α above 0.7, which indicates a good internal consistency of the questionnaire.

**Table 3:** Mean score, internal consistency, and interclass correlation coefficient values of items. *Other practices (items=25) were used for qualitative information, not analyzed for internal consistency. ICC: intraclass correlation coefficient.

Subscale	Items	Mean ± SD	Cronbach’s α	ICC (mean)	ICC range
Knowledge	33	16.14 ± 7.07	0.980	0.845	0.705-0.987
Attitude	21	52 ± 5.51	0.840	0.867	0.775-0.997
Practices					
Dietary	55	129 ± 48.97	0.930	0.769	0.701-0.945
Other*	25	-	-	-	-

The final 134 items in the questionnaire were administered twice to children at intervals of 15 days. For knowledge and attitude items, ICC estimates ranged from 0.705 to 0.987 (mean 0.845), 0.775 to 0.997 (mean 0.867), respectively, whereas ICC estimates ranged for practice items from 0.701 to 0.945 (mean 0.769) (Table 3).

So, based on the results of validity and reliability, a 134-item questionnaire consisting of 33 knowledge items, 21 attitudes, and 80 practice items (55 dietary practices and 25 other practices related to micronutrients) was finalized (the questionnaire is given in the Appendix, Table 4).

## Discussion

This study was carried out to develop and validate a questionnaire for assessing the knowledge, attitude, and practice of micronutrients among adolescents. Measures of validity, such as content validity, and measures of reliability, such as internal consistency and test-retest, were used to develop the questionnaire. We could not find similar studies that developed and validated KAP questionnaires on micronutrients among adolescent girls. Similar to the present study, another study was conducted by Konapur et al. (2019) among literate mothers of school-age children, which developed and validated KAP questionnaires on micronutrients using a mixed method approach and they used similar methods such as content validity, internal consistency, and test-retest reliability [15]. Another study by Augustine et al. (2011) assessed psychometric validation of only knowledge questionnaires on micronutrients among 15-to 19-year-old adolescent boys using similar methods [16].

A review of the literature shows that most of the studies have developed questionnaires on general nutrition knowledge, attitude, and practices, and these studies validated questionnaires using similar techniques, which we have used in the present study. These questionnaires were mainly developed for different study populations, such as parents of children and adolescents [17] elderly [18], 13-14-year-old female adolescents [19], and school teachers [20]. However, some studies developed the KAP questionnaires on general nutrition and micronutrients but have not been validated [21-25]. Some studies evaluated questionnaires using pilot testing [24], whereas some studies have just mentioned pre-tested questionnaire was used, but they did not mention the steps they used for the development and validation of the questionnaire [23].

Most of the studies have developed KAP on a single micronutrient, such as vitamin D [13] and iron or iron deficiency anemia [26-28]. In the present study, we have focused KAP assessment of different micronutrients (vitamin A, vitamin C, vitamin B12, folate, vitamin D, calcium, iron, and zinc).

In the present study, we observed that knowledge of micronutrients among adolescent girls was very poor. It was also observed that healthy eating practices were not properly followed by adolescent girls. This study revealed that there is a lack of knowledge among adolescent girls about micronutrients, which may be due to a lack of awareness or because they would not have come across the in-depth nutritional content. These findings suggest that there is a need to make efforts to enhance adolescents’ nutrition knowledge so that they can make the necessary changes in behavior to adopt healthy lifestyle habits and good dietary practices. Through this questionnaire, knowledge, attitudes, and practices can be assessed, and based on findings, we can plan the intervention program for the target group. It can also be used to assess the impact of nutrition education intervention programs that mainly focus on improving nutrition status and creating positive attitudes towards healthy eating practices among girls.

The strength of the present study is we developed a valid and reliable tool to measure KAP related to micronutrients. As mentioned above, there are limited studies on KAP towards micronutrients, and most of the studies are on nutrition knowledge. Second, the present study was conducted in Mumbai, where due to rapid urbanization is linked to changes in lifestyle practices and easy access to unhealthy food [29]. The participants in the present study were in the stage of adolescence, in which unhealthy eating behaviors and inadequate intake of fruits and vegetables are reported in the literature [30]. So, it is very important to know their knowledge, attitude, and practices towards micronutrients. Lastly, we have included both open and close-ended questions in the questionnaire. The inclusion of an open-ended questionnaire in the knowledge subscale will be helpful to get the real responses given by participants.

However, it is important to note that our study has limitations in generalizability because we have used a purposive sampling method and a study conducted in the urban setting to assess the psychometric properties of the questionnaire. Further studies with different diverse settings are needed to validate this questionnaire.

## Conclusions

The result of the present study provides clear evidence of the validity and reliability of the questionnaire, which can be used to assess knowledge, attitudes, and practices on micronutrients among adolescent girls. These findings will have significance for structuring the behavioral change intervention program based on the transtheoretical model. This study recommends the implementation of nutrition intervention programs for educational institutes to create awareness and improve the knowledge of micronutrients regularly, to change the eating behavior of adolescent girls. This behavioral change will be helpful to improve their micronutrient status and prevent them from micronutrient deficiencies. This tool can also be useful to policymakers in developing effective nutrition education intervention programs.

## Appendices

**Table 4:** Knowledge, attitude, and practice questionnaire on micronutrient intake.

Sr.no.	Questions	Responses										
Knowledge												
1)	Are you aware of the nutrients in food?	Yes	No									
	If yes, name them											
2)	Have you heard about the micronutrients	Yes	No									
	If yes, name them											
3)	Do you know the sources of the following nutrients	Name of the sources	Don’t know									
3a)	Vitamin A											
3b)	Vitamin B12											
3c)	Vitamin D											
3d)	Vitamin C											
3e)	Folate											
3f)	Iron											
3g)	Zinc											
3h)	Calcium											
4)	Do you know any deficiency symptoms if we don’t consume adequate nutrients?	Name of the symptoms	Don’t know									
4a)	Vitamin A											
4b)	Vitamin B12											
4c)	Vitamin D											
4d)	Vitamin C											
4e)	Folate											
4f)	Iron											
4g)	Zinc											
4h)	Calcium											
5)	Do you know the causes of deficiency of the following nutrients?	Mention the causes	Don’t know									
5a)	Vitamin A											
5b)	Vitamin B12											
5c)	Vitamin D											
5d)	Vitamin C											
5e)	Folate											
5f)	Iron											
5g)	Zinc											
5h)	Calcium											
6)	Do you know how these deficiencies can be prevented?	Yes	No									
	If yes, mention the ways to prevent the same											
7)	Do you know what Hemoglobin is?	Yes	No									
	If yes, what is the normal level of Hemoglobin											
8)	Do you know which food helps in iron absorption?	Yes	No									
	If yes, name them											
9)	Do you know foods which decrease iron absorption?	Yes	No									
	If yes, name them											
10)	Do you know which vitamin increases calcium absorption	Yes	No									
	If yes, name them											
11)	Do you know about micronutrient supplements available in the market	Yes	No									
	If yes, name a few											
12)	Do you know what is the best time to get sun exposure for vitamin D?	Yes	No									
	If yes, mention											
Attitude												
1)	Do you think you may have deficiency of following nutrients?	Agree	Undecided		Disagree							
1a)	Vitamin A											
1b)	Vitamin B12											
1c)	Vitamin D											
1d)	Vitamin C											
1e)	Folate											
1f)	Iron											
1g)	Zinc											
1h)	Calcium											
2)	Do you think micronutrient deficiency is harmful for health	Agree	Undecided		Disagree							
3)	It is good for you to consume variety of foods every day	Agree	Undecided		Disagree							
4)	It is not difficult for you to consume different types of food every day	Agree	Undecided		Disagree							
5)	Knowing more about micronutrients is good for your heath	Agree	Undecided		Disagree							
6)	Daily we should eat fruits and vegetables	Agree	Undecided		Disagree							
7)	Daily we should consume milk and milk products	Agree	Undecided		Disagree							
8)	We should consume meat on a regular basis	Agree	Undecided		Disagree							
9)	We should consume fish on a regular basis	Agree	Undecided		Disagree							
10)	We should consume egg and chicken on a regular basis	Agree	Undecided		Disagree							
11)	Daily we need to take Sun exposure	Agree	Undecided		Disagree							
12)	Personal hygiene is important to prevent micronutrient deficiencies	Agree	Undecided		Disagree							
13)	Nutrition awareness and education is important to prevent and control the nutritional deficiencies	Agree	Undecided		Disagree							
14)	Micronutrient supplementation is important to prevent and control deficiencies	Agree	Undecided		Disagree							
Other practices												
1)	Have you ever gone for laboratory estimations of micronutrients	Yes	No									
	Yes, mention											
2)	Do you take any of the following micronutrient supplements?	Yes	No									
2a)	Vitamin A											
2b)	Vitamin B12											
2c)	Vitamin D											
2d)	Vitamin C											
2e)	Folate											
2f)	Iron											
2g)	Zinc											
2h)	Calcium											
3)	Have you taken deworming tablets in the past six months?	Yes	No									
4)	Do you or your household members use iron pot/vessel for cooking?	Yes	No									
5)	What kind of clothing you generally wear?	Western dress OR Indian dress (salwar kameez/saree)	Hijab and abaya (face and palms uncovered)		Niqab (face covered)							
6)	When do you usually eat the following foods?	Taken with the meal	Time gap before the meal	Time gap after the meal		Don’t know/no answer						
6a)	Lemon											
6b)	Amla											
6c)	Guava											
6d)	Papaya											
6e)	Orange											
6f)	Sweet Lime											
6g)	Tea											
6h)	Coffee											
7)	What are your timings and duration of sun exposure?	None	15 min		30 min	1 h	2 h					
7a)	7 am–11 am											
7b)	11 am –3 pm											
7c)	3 pm –7 pm											
8)	Do you take precautionary measures during sun exposure?	Yes	No									
9)	What is your primary sun protection?	Umbrella	Hats		Scarf	Sunscreen lotion		Other				
Dietary practices: How often do you eat the following foods?												
	Food items	Daily	Two to three times in the week		Once in week	Fortnightly		Monthly		Rarely	Never	Rarely
1	Rice flakes											
2	Bajra											
3	Ragi											
4	Rajgeera											
5	Jowar											
6	Dals											
7	Beans											
8	Milk											
9	Curd											
10	Paneer											
11	Cheese											
12	Lassi											
13	Buttermilk											
14	Milkshake											
15	Lal Math											
16	Amaranth leaves											
17	Colocasia Leaves											
18	Drumstick Leaves											
19	Fenugreek Leaves											
20	Radish Leaves											
21	Shepu											
22	Spinach											
23	Spring onion											
24	Beet root											
25	Carrot											
26	Sweet potato											
27	Yam (Suran)											
28	Capsicum											
29	Lal bhopala											
30	Tomato (raw/ripe)											
31	Cabbage											
32	Cauliflower											
33	Amla											
34	Guava											
35	Lime, sweet											
36	Mango, ripe											
37	Melon, musk											
38	Melon, water											
39	Orange											
40	Papaya											
41	Chicken											
42	Mutton											
43	Fish											
44	Egg											
45	Til											
46	Flax seeds/Alsi											
47	Ground nuts											
48	Almonds											
49	Cashew											
50	Walnuts											
51	Pistachio											
52	Fig/Anjeer											
53	Garden Cress seeds (Halim)											
54	Raisins											
55	Pumpkin seeds											
